# Anaesthesia-related complications after endovascular therapy for acute ischaemic stroke under general anaesthesia with early extubation: A single-centre retrospective cohort study

**DOI:** 10.1016/j.bjao.2026.100547

**Published:** 2026-04-10

**Authors:** Ida Hartor Jensen, Andreas G. Damsbo, Jan B. Valentin, Sissel Marie Stokvad, Anne B. Behrndtz, Rolf A. Blauenfeldt, Ronni Mikkelsen, Claus Z. Simonsen, Mads Rasmussen

**Affiliations:** 1 Department of Anaesthesiology, Section of Neuroanaesthesia, Aarhus University Hospital, Aarhus, Denmark; 2 Department of Neurology, Aarhus University Hospital, Aarhus, Denmark; 3 Department of Neurology, Regional Hospital Goedstrup, Goedstrup, Denmark; 4 Danish Centre for Health Services Research, Aalborg University, Aalborg, Denmark; 5 Department of Clinical Medicine, Faculty of Health, Aarhus University, Aarhus, Denmark; 6 Department of Radiology, Aarhus University Hospital, Aarhus, Denmark

**Keywords:** acute ischaemic stroke, complications, endovascular therapy, extubation, general anaesthesia, intensive care, outcomes, predictors

## Abstract

**Background:**

The optimal anaesthetic strategy for endovascular therapy (EVT) in acute ischaemic stroke (AIS) remains debated. We evaluated the incidence of anaesthesia-related complications, their predictors, and the associated clinical outcomes of a standardised general anaesthesia (GA) protocol.

**Methods:**

We retrospectively analysed patients undergoing EVT for AIS under GA over a 4-yr period. Patients were classified as having a complicated or uncomplicated course based on anaesthesia-related complications within 24 h post-EVT, defined as transfer from the angiography suite to the intensive care unit (ICU), prolonged stay at the post anaesthesia care unit (PACU), PACU-to-ICU transfer, or ICU transfer from the stroke ward. Predictors of a complicated course were analysed. The primary outcome was complication incidence. Secondary outcomes were 3-month functional outcome (modified Rankin Scale [mRS] 0–2 *vs* 3–6), change in 24-h National Institutes of Health Stroke Scale (NIHSS), pneumonia rate, and 3-month mortality.

**Results:**

Of 734 patients, 133 (18%) experienced an anaesthesia-related complication and had a complicated course. Admission NIHSS, younger age, and posterior circulation occlusion were the strongest predictors. These patients had smaller 24-h NIHSS improvement (mean difference 5.4, 95% confidence interval [CI] 4.1–6.7), higher pneumonia risk (odd ratio [OR]: 2.72, 95% CI: 1.66–4.48), poorer functional outcomes (mRS: 3–6; OR: 3.65, 95% CI: 2.15–6.20), and higher mortality (OR: 4.20, 95% CI: 2.45–7.21) compared with those with an uncomplicated course.

**Conclusions:**

Complications occurred in 133 (18%) patients and were more frequent in those with higher NIHSS scores and posterior circulation occlusions. Patients with a complicated course had poorer functional outcomes at 3 months.

Endovascular therapy (EVT) is the standard treatment for acute ischaemic stroke (AIS) as a result of large vessel occlusion (LVO) in both the anterior and posterior circulation.^1^, ^2^, ^3^, ^4^ However, the optimal anaesthetic and perioperative approach during EVT remains debated.^4^^,^^5^ Single-centre randomised controlled trials, and meta-analyses suggest that EVT under general anaesthesia (GA), with controlled blood pressure (BP), may improve functional outcomes compared with conscious sedation (CS) or local anaesthesia.^6^, ^7^, ^8^, ^9^ GA with endotracheal intubation ensures airway protection and patient immobility, contributing to higher reperfusion rates, but is also linked to complications such as haemodynamic instability, procedural delays, and extubation challenges, especially in patients with comorbidities or extensive cerebral injury.^10^

The number of patients undergoing EVT is increasing as a result of broader eligibility criteria, growing evidence of efficacy, and an ageing population.^11^, ^12^, ^13^, ^14^ As a result, the use of GA may increase, potentially increasing the demand on intensive care unit (ICU) resources and placing a strain on the post anaesthesia care unit (PACU) and stroke ward, as patients following EVT have a higher risk of respiratory and haemodynamic complications.^15^, ^16^, ^17^, ^18^, ^19^, ^20^ Post-procedural extubation strategies vary across institutions, ranging from immediate extubation in the angiography suite to delayed extubation in the ICU.^15^, ^16^, ^17^, ^18^^,^^21^ However, no guidelines currently define the optimal timing for extubation in this context.^4^

Since 2019, our institution has implemented GA with early extubation in the angiography suite as the standard approach for EVT. Under this standardised pathway, patients were extubated in the angiography suite and transferred to the PACU according to clinical status. In this retrospective cohort study, we describe the incidence and characteristics of anaesthesia-related complications within 24 h post-EVT, including events such as extubation failure leading to ICU admission or prolonged PACU stay. We further characterise clinical factors predicting these events and report the corresponding functional outcomes.

## Methods

### Data availability

Given the prevailing Danish law, the authors are not permitted to share the data or grant access to the data.

### Study design and setting

We performed a retrospective cohort study of patients undergoing thrombectomy at Aarhus University Hospital, Aarhus, Denmark.

### Participants and eligibility criteria

We included all adult patients (18 yr of age or older) who presented with LVO in the anterior or posterior circulation and who underwent EVT under GA with intubation between 1 January 2020 and 31 December 2023. We excluded patients younger than 18 years, patients with missing anaesthesia, PACU, or ICU charts, and those who underwent endovascular therapy under CS or local anaesthesia.

### Ethical approval and reporting standards

The study was approved by the management of Aarhus University Hospital, Denmark (approval no 1-49-72-4-18, granted 5 March 2024), and the involved departments. According to Danish law, approval from a national ethics committee and the Danish Data Protection Authority is not required for a quality improvement project; thus, patient consent was waived by the local Data Protection Authority. The study and findings are reported according to the Strengthening the Reporting of Observational Studies in Epidemiology (STROBE) statement for observational studies.^22^

### EVT-GA protocol

**Fig 1:**
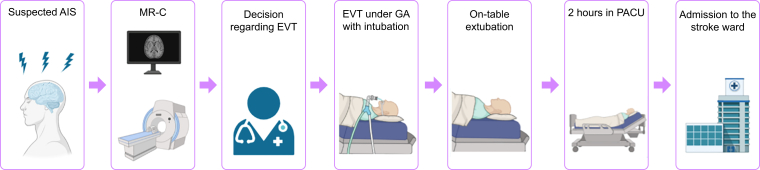
The EVT-GA protocol at Aarhus University Hospital, Aarhus, Denmark. The EVT-GA protocol outlines the management of patients with an uncomplicated course. Patients with suspected AIS are admitted to the EVT-centre and undergo MRI using a dedicated stroke protocol. For transfer patients from primary stroke centres, MRI is performed only if there is a change in neurological status. The decision to proceed with EVT is made collaboratively by a neurovascular neurologist and an interventional neuroradiologist. The procedure is performed under GA in the angiography suite, followed by immediate post-procedure extubation and transfer to the PACU for a standardised 2-h observation period. After PACU monitoring, patients are transferred directly to the stroke ward. AIS, acute ischaemic stroke; EVT, endovascular therapy; GA, general anaesthesia; MR-C, magnetic resonance imaging of the brain; PACU, post-anaesthesia care unit.

Figure 1 shows the EVT-GA protocol at Aarhus University Hospital. Patients treated with EVT are either directly admitted to our comprehensive EVT-centre or referred from two primary stroke centres.

The decision to proceed with EVT was made collaboratively by a neurovascular neurologist and an interventional neuroradiologist. Following the decision, patients were transferred directly to the angiography suite.

General anaesthesia with endotracheal intubation was induced and maintained using intravenous anaesthetics (propofol and remifentanil).^6^^,^^10^ Intra-procedural BP is carefully managed, targeting a systolic blood pressure (SBP) of 140–180 mmHg and a mean arterial blood pressure (MABP) > 70 mmHg.^23^, ^24^, ^25^ Normoventilation was maintained throughout the procedure. All patients were assessed for immediate extubation in the angiography suite upon completion of EVT.

Anaesthesia care during EVT was routinely provided by specialist neuroanaesthesiologists and nurse anaesthetists with subspecialty training in neuroanaesthesia.

Following extubation, patients were transferred to the PACU for a standardised 2-h observation period. During this time, heart rate, BP, oxygen saturation, and neurological status, including level of consciousness, were continuously monitored by nurses specialised in post-anaesthesia care.

In reperfused patients, SBP was maintained < 160 mmHg, and < 180 mmHg in non-reperfused patients according to institutional guidelines. After PACU observation, patients were transferred to the stroke ward.

### Definition of complicated *vs* uncomplicated course

A complicated course was defined as any post-EVT anaesthesia-related complication—respiratory, haemodynamic, neurological, organisational, or other—that resulted in: (1) direct ICU admission from the angiography suite; (2) prolonged PACU stay (>2 h) or ICU transfer from the PACU; or (3) ICU transfer from the stroke ward within 24 h post-EVT. Patients without anaesthesia-related complications within 24 h post-EVT were classified as having an uncomplicated course. Figure 1 illustrates the EVT-GA pathway for patients with an uncomplicated course.

### Outcome measures

Only one primary outcome was prespecified, while all other outcomes were considered secondary and exploratory. The primary outcome measure was the proportion of patients with a complicated *vs* an uncomplicated course. Secondary outcomes were 3-month functional outcome (modified Rankin Scale [mRS]: 0–2 *vs* 3–6), change in National Institutes of Health Stroke Scale (NIHSS) score at 24 h (defined as the difference between admission and 24-h NIHSS), incidence of pneumonia requiring treatment, and 3-month mortality.

### Data sources and management

Clinical data were extracted from patient records, including anaesthesia, PACU, and ICU charts. This included patient characteristics and indications for ICU admission directly from the angiography suite as a result of early extubation failure—defined as either a clinical decision not to extubate immediately after the procedure because of medical instability, neurological impairment, other concerns, or re-intubation occurring immediately following an initial extubation attempt. Other ICU admission criteria included reduced consciousness, respiratory insufficiency, haemodynamic instability, or organisational/logistical concerns (no space in PACU, need for extended monitoring). Characteristics and causes of prolonged PACU stay were documented and defined as exceeding 2 h or not meeting institutional discharge criteria, including transfers from PACU to ICU, and ICU admissions from the stroke ward.

As the use of GA may increase the likelihood of pneumonia,^17^ the proportion of patients treated with antibiotics for suspected pneumonia was recorded, with pneumonia defined as initiation of antibiotic therapy during the stroke unit stay for suspected or confirmed lower respiratory tract infection.

Post-extubation physiological parameters in PACU, including SBP, diastolic blood pressure (DBP), MABP, heart rate, and oxygen saturation, were documented along with PACU and ICU admission and discharge times.

All data were entered into a REDCap (Vanderbilt University, Nashville, TN, USA) database and merged with the Aarhus University Hospital Stroke Database. This database includes functional outcomes (3-month mRS, range 0–6, with higher scores indicating greater disability), demographics, vascular risk factors, intravenous thrombolysis (IV tPA) use, admission and 24-h NIHSS (range 0–42) scores, magnetic resonance imaging (MRI) findings (occlusion site), reperfusion status (modified Thrombolysis in Cerebral Infarction [mTICI] score), and procedural time metrics (onset-to-reperfusion, MRI-to-angiography suite, puncture-to-reperfusion), as well as total hospitalisation duration.

Patient follow-up with mRS assessment was performed by an mRS-certified study nurse or neurovascular neurologist, and the mTICI score was determined by the interventional neuroradiologist performing the EVT procedure. These assessments were performed independently of this study, and the assessors were therefore effectively blinded to the study endpoints.

### Statistical analysis

Descriptive and comparative analyses were performed between patients having a complicated or uncomplicated course.

Missing data are reported for each variable, and an overall missing-value assessment was performed across all variables. Missing data were handled using multiple imputations by chained equations.^26^ For each regression model, the relevant dataset was selected and imputed based on the selected covariates. Clinical outcome variables were not imputed in any analysis. All imputations were based on sets of 10 imputations, each generated from 10 iterations.

The groups of patients with complicated and uncomplicated pathways were compared based on the amount of missing data for each patient characteristic and outcome parameter to assess the potential risk of selection bias attributable to missingness. Regardless of the results, missing data were handled under the assumption of 'missing at random', meaning that any potential selection bias arising from missingness was explained by observed parameters.

Patient- and procedure-related factors were assessed as potential predictors of a complicated course using multivariable logistic regression analyses. The multivariable analyses were performed on both complete and imputed datasets.

To interpret the contribution of each specific variable, Shapley additive explanation (SHAP) values were calculated, with the magnitude of the SHAP value indicating the strength of the predictive effect.^27^ Prediction model performance was evaluated using 10-fold cross-validation, with receiver operating characteristic (ROC) curves, area under the curve (AUC), and calibration curves estimated for both datasets. The variables included in the prediction model were selected based on availability and because they are established predictors of stroke severity.

Regression model assumptions were evaluated using standard diagnostic tests, including assessment of linearity, homoscedasticity, and residual distributions. Multicollinearity was examined using variance inflation factors. To estimate the risk of chance findings in the prediction analysis, we performed a post hoc power assessment.^28^^,^^29^ Given the 18 included parameters and an assumed AUC of 0.7, the required sample size to adequately minimise the risk of chance findings would be 2087 patients.

To assess outcomes of patients experiencing a complicated course, binary outcomes were analysed using logistic regression, both unadjusted and adjusted for confounders, including continuous variables (age, recorded in years; pre-stroke mRS, assessed on a 0–6 scale; mTICI score, graded 0–3 based on final angiographic reperfusion; NIHSS score, assessed on a 0–42 scale at admission; and time from onset to angiography suite arrival, recorded in minutes), dichotomous variables (sex, coded as male/female; and IV tPA treatment, coded as yes/no), and categorical variables (occlusion location, classified according to standard vascular territories based on pre-procedural imaging). These variables are established predictors of stroke severity and outcomes and may influence both the risk of a complicated course and clinical endpoints; adjusting for them minimises confounding bias. Changes in NIHSS score were analysed using a mixed model for repeated measures (MMRM), as implemented in R with the MMRM package.^30^ This analysis was adjusted for the covariates listed above, except baseline NIHSS. Adjusted analyses were performed on both complete and imputed datasets. Statistical significance was set at *P*<0.05. All analyses were conducted using R software.

## Results

**Fig 2:**
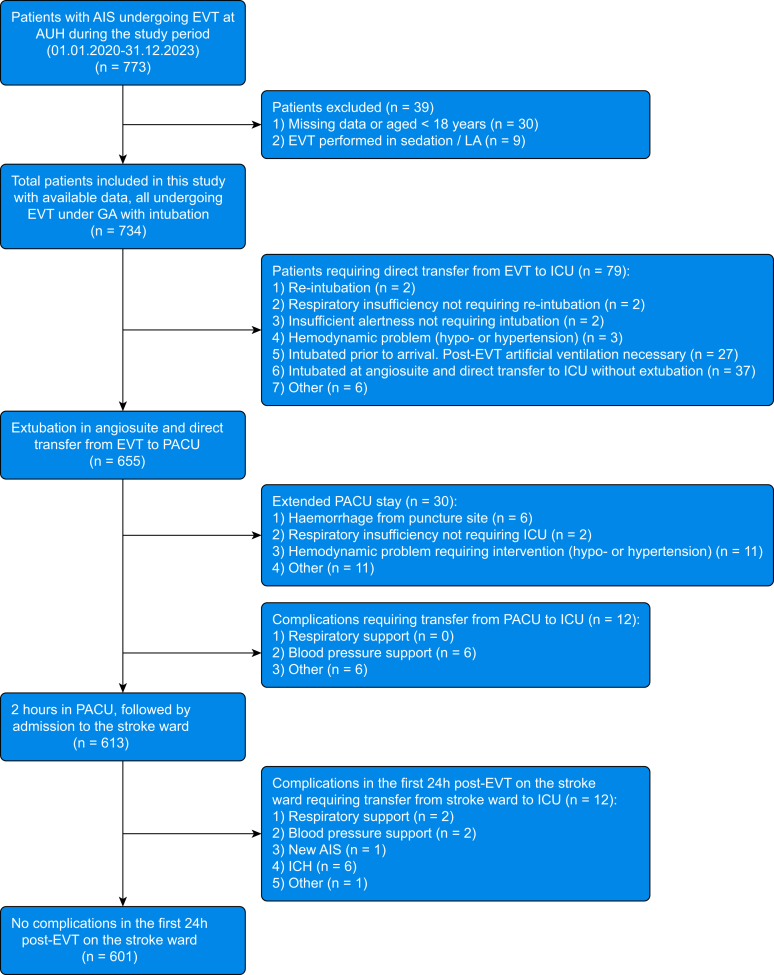
Flow chart of patients included in the analysis. From 1 January 2020 to 31 December 2023, a total of 773 patients underwent EVT for AIS at Aarhus University Hospital, Aarhus, Denmark. Thirty-nine patients were excluded because of missing data or because the procedure was performed under local anaesthesia or conscious sedation. Among the remaining patients, 133 (18%) experienced post-EVT anaesthesia-related complications resulting in a complicated course within the first 24 h post-EVT. The remaining 601 patients (82%) had an uneventful post-EVT recovery. AIS, acute ischaemic stroke; AUH, Aarhus University Hospital; EVT, endovascular therapy; GA, general anaesthesia; ICH, intracerebral haemorrhage; ICU, intensive care unit; LA, local anaesthesia; PACU, post-anaesthesia care unit.

From 1 January 2020 to 31 December 2023, a total of 773 patients underwent EVT for AIS at Aarhus University Hospital (Fig. 2). Thirty-nine (5%) patients were excluded because of missing data or because local anaesthesia or CS was used in cases deemed unfit for GA. Thus, 734 patients were included in the analysis. The median age was 73 yrs (interquartile range [IQR]: 62–80), 317 (43%) were female, median NIHSS at admission was 15 (IQR: 8–19), median 24-h NIHSS was 7 (IQR: 3–16), and 604 patients had LVO occlusion in the anterior circulation (83%) (Table 2).

**Table 1:** Categorisation of complications leading to a complicated course. Respiratory (e.g. extubation failure, need for ventilatory support, airway interventions); Haemodynamic (e.g. vasopressor escalation, severe hypotension, arrhythmias); Neurological (e.g. decreased level of consciousness prompting ICU transfer); Organisational/Logistical (e.g. prolonged PACU stay as a result of resource limitations, unplanned ICU admission primarily for monitoring). ICU, intensive care unit; PACU, post-anaesthesia care unit.

Complications	Number of patients, *n* (%)
**Respiratory**	42 (31.6%)
**Haemodynamic**	41 (30.8%)
**Neurological**	41 (30.8%)
**Organisational / logistical**	9 (6.8%)

**Table 2:** Baseline characteristics. AB, antibiotics; AIS, acute ischaemic stroke; AMI, acute myocardial infarction; EVT, endovascular therapy; ICU, intensive care unit; IV tPA, intravenous thrombolysis; IQR, interquartile range; MRI, magnetic resonance imaging; mRS, modified Rankin Scale; mTICI, modified thrombolysis in cerebral infarction score; NIHSS, National Institutes of Health Stroke Score; PACU, post-anaesthesia care unit; PAD, peripheral artery disease; TIA, transient ischaemic attack.

Characteristics	Overall *n*=734	Complicated course *n*=133	Uncomplicated course *n*=601
**Age, median (IQR), yrs**	73 (62–80)	69 (60–77)	74 (64–81)
**Female sex, *n* (%)**	317 (43%)	60 (45%)	257 (43%)
**Admission NIHSS score, median (IQR)**	15 (8–19)	18 (9–24)	15 (8–19)
Missing data	20	6	14
**Pre-stroke mRS, *n* (%)**			
0	522 (72%)	93 (70%)	429 (73%)
1	93 (13%)	10 (7.5%)	83 (14%)
2	74 (10%)	17 (13%)	57 (9.7%)
3	23 (3.2%)	10 (7.5%)	13 (2.2%)
4	7 (1.0%)	1 (0.8%)	6 (1.0%)
5	2 (0.3%)	2 (1.5%)	0 (0%)
Missing data	13	0	13
**Previous AIS, *n* (%)**	87 (12%)	17 (13%)	70 (12%)
Missing data	22	2	20
**Previous TIA, *n* (%)**	46 (6.4%)	6 (4.5%)	40 (6.8%)
Missing data	10	0	10
**Arterial hypertension, *n* (%)**	426 (59%)	86 (66%)	340 (57%)
Missing data	11	2	9
**Diabetes mellitus, *n* (%)**	107 (15%)	24 (18%)	83 (14%)
Missing data	12	1	11
**Atrial fibrillation, *n* (%)**	203 (28%)	31 (23%)	172 (29%)
Missing data	11	1	10
**Previous AMI, *n* (%)**	73 (10%)	13 (9.9%)	60 (10%)
Missing data	16	2	14
**PAD, *n* (%)**	29 (4.0%)	7 (5.3%)	22 (3.7%)
Missing data	12	0	12
**Smoking, *n* (%)**			
Smoking	176 (28%)	35 (32%)	141 (27%)
Missing data	101	24	77
**High alcohol consumption, *n* (%)**	66 (10%)	13 (12%)	53 (10.0%)
Missing data	97	27	70
**Location of vascular occlusion, *n* (%)**			
Anterior	604 (83%)	86 (65%)	518 (86%)
None	35 (4.8%)	5 (3.8%)	30 (5.0%)
Posterior	92 (13%)	41 (31%)	51 (8.5%)
Missing data	3	1	2
**IV tPA before EVT, *n* (%)**	316 (43%)	46 (35%)	270 (45%)
Missing data	17	3	14
**mTICI score, *n* (%)**			
0	36 (5.0%)	12 (9.2%)	24 (4.1%)
1	2 (0.3%)	1 (0.8%)	1 (0.2%)
2a	11 (1.5%)	4 (3.1%)	7 (1.2%)
2b	189 (26%)	40 (31%)	149 (25%)
2c	43 (6.0%)	3 (2.3%)	40 (6.8%)
3	438 (61%)	71 (54%)	367 (62%)
Missing data	15	2	13
**Successful reperfusion (mTICI score 2b-3), *n* (%)**	670 (93%)	114 (87%)	556 (95%)
Missing data	15	2	13
**Median 24-hr NIHSS, *n* (%)**	7 (3, 16)	17 (7, 25)	6 (2, 13)
Missing data	71	25	46
**AB-treated pneumonia, *n* (%)**	138 (19%)	46 (35%)	92 (15%)
Missing data	5	3	2
**Length of stay, PACU, median (IQR), hrs**	2.08 (1.92–2.33)	3.71 (2.18–6.17)	2.08 (1.92–2.25)
Missing data	79	79	0
**Length of stay, ICU, median (IQR), h**	23 (13–51)	23 (13–51)	NA (NA–NA)
Missing data	655	54	601
**Onset to reperfusion, median (IQR), minutes**	276 (185–524)	312 (241–626)	266 (174–500)
Missing data	136	30	106
**MRI to arrival angiosuite, median (IQR), minutes**	35 (21–52)	41 (21–59)	34 (22–51)
Missing data	227	47	180
**Arrival angiosuite to groin, median (IQR), minutes**	17 (14–20)	17 (12–22)	17 (14–20)
Missing data	100	21	79
**Arrival angiosuite to reperfusion, median (IQR), minutes**	43 (32–61)	47 (32–71)	42 (32–57)
Missing data	130	29	101
**Arrival angiosuite to end of procedure, median (IQR), minutes**	61 (42–90)	79 (50–115)	58 (42–83)
Missing data	78	13	65
**Length of hospital admission, median (IQR), days**	3.5 (1.6–7.4)	4.5 (1.9–10.5)	3.3 (1.5–6.5)

**Table 3:** Outcomes. Dichotomous outcomes were analysed using logistic regression, both unadjusted and adjusted for age, sex, pre-stroke mRS, mTICI score, NIHSS score, occlusion location, IV tPA treatment, and time from onset to angiography suite arrival. Multivariable analyses were performed on both complete and imputed datasets, and results are presented as OR with 95% CI. Changes in NIHSS score (defined as the difference between admission NIHSS and 24-h NIHSS post-EVT) were analysed using a mixed model for repeated measures, adjusted for the covariates listed above except baseline NIHSS. Results are presented as the mean change in NIHSS score. Patients with a complicated course demonstrated markedly smaller improvements in NIHSS. 95% CI, 95% confidence interval; AB, antibiotics; IV tPA, intravenous thrombolysis; mRS, modified Rankin Scale; mTICI, modified Thrombolysis in Cerebral Infarction score; NIHSS, National Institutes of Health Stroke Score; OR, odds ratio.

Outcome	Overall *n*=734	Complicated course *n*=133	Uncomplicated course *n*=601	Unadjusted OR (95% CI)	Adjusted OR (95% CI)	Adjusted imputed OR (95% CI)
**Dichotomised 3 months mRS, *n* (%)**				4.43 (2.89–6.98)	4.35 (2.47–7.86)	3.65 (2.15–6.20)
0–2	365 (51)	30 (23)	335 (57)			
3–6	352 (49)	100 (77)	252 (43)			
Missing data	17	3	14			
**AB-treated pneumonia, *n* (%)**	138 (19)	46 (35)	92 (15)	3.02 (1.97–4.59)	3.14 (1.83–5.36)	2.72 (1.66–4.48)
Missing data	5	3	2			
**Mortality within 3 months, *n* (%)**	124 (17)	53 (41)	71 (12)	5.00 (3.26–7.69)	3.94 (2.16–7.22)	4.20 (2.45–7.21)
Missing data	17	3	14			

				**Unadjusted**	**Adjusted**	**Adjusted imputed**

**NIHSS score change, mean difference (95% CI)**				6.0 (4.8–7.2)	5.8 (4.4–7.1)	5.4 (4.1–6.7)
Missing data	71	25	46			

A total of 133 patients (18%) developed anaesthesia-related complications within 24 h post-EVT, resulting in a complicated course. A categorised presentation of these complications is shown in Table 1. The consequences included direct ICU transfer from the angiography suite (*n*=79, 11%), ICU transfer from the PACU (*n*=12, 1.5%), prolonged PACU stay (*n*=30, 4%), and ICU transfer from the stroke ward within 24 h (*n*=12, 1.5%). Extubation failure occurred in 66 patients (9% of all cases), accounting for 83.5% of direct ICU admissions. This included immediate re-intubation in the angiography suite (*n*=2), pre-hospital intubation with patients deemed unfit for extubation (*n*=27), and pre-procedural intubation in the angiography suite followed by unsuitability for extubation (*n*=37). Detailed reasons for unsuitability are provided in Supplementary Table 1. The remaining 601 patients (82%) had an uncomplicated course within 24 h post-EVT (Fig. 2). The missing data analysis is presented in Supplementary Table 2.

### Baseline characteristics

Baseline characteristics are shown in Table 2. Patients experiencing a complicated course had a lower age, higher admission and 24-h NIHSS, higher pre-stroke mRS, more frequent occlusion of the posterior circulation, a lower rate of successful reperfusion (mTICI 2b-3), and fewer patients received IV tPA. The length of stay in PACU, onset-to-reperfusion, arrival at angiography suite-to-reperfusion, arrival at angiography suite-to-end of procedure, and length of hospital admission were longer. Sex and comorbidities did not influence the risk of a complicated course.

### Key-predictors of a complicated course

**Fig 3:**
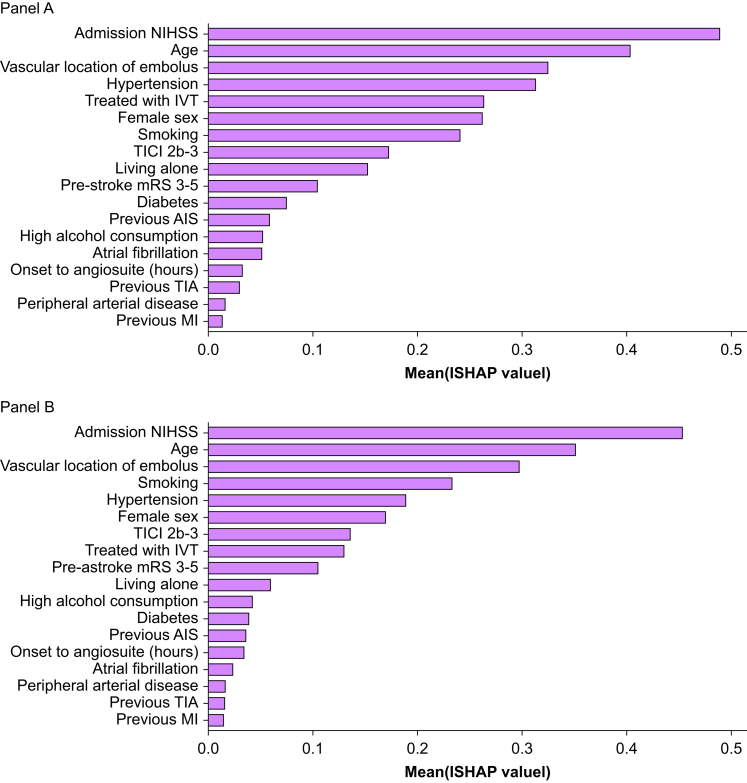
SHAP values from the multivariate regression analysis. The values indicate the relative importance of predictors. SHAP analysis of both observed (panel A, upper) and imputed datasets (Panel B, lower) identified high admission NIHSS, younger age, and vascular occlusion location (posterior occlusion) as the strongest predictors of a complicated course. AIS, acute ischaemic stroke; IVT, intravenous thrombolysis; MI, myocardial infarction; mRS, modified Rankin Scale; NIHSS, National Institutes of Health Stroke Score; SHAP, Shapley additive explanations; TIA, transient ischaemic attack; TICI, Thrombolysis in Cerebral Infarction score.

SHAP analysis of both observed and imputed datasets identified markers of clinical severity, including high admission NIHSS, younger age, and vascular occlusion location (posterior circulation), as the strongest predictors of a complicated course (Fig. 3).

Hypertension, smoking, female sex, IV tPA, and mTICI status contributed moderately, while pre-stroke disability, diabetes, atrial fibrillation, previous ischaemic events, lifestyle factors, and procedural characteristics had only a minor impact. Findings were consistent across complete and imputed datasets, with slight variations in ranking (notably smoking and IV tPA), indicating overall robustness of the model. The coefficients from the regression model are shown in Supplementary Table 3.

### Model performance in predicting a complicated course

#### ROC curves

Supplementary Figure 1 displays ROC curves illustrating model performance in predicting a complicated course. Panel A shows the ROC curve for the observed dataset (AUC=0.69), and Panel B for the imputed dataset (AUC=0.71). Both values indicate moderate discriminative ability.

#### Calibration plots

Calibration plots for complete cases and imputed data are shown in Supplementary Figure 2. In the complete case dataset, the model demonstrated reasonable agreement between predicted and observed risks at lower probabilities but showed miscalibration in the mid-range, with a slight overestimation of predicted risks > 50%. In contrast, the imputed data analysis yielded smoother calibration curves with closer alignment to the ideal line. Overall, calibration was improved following imputation compared with the complete case analysis.

We assessed all regression models and found no violations of assumptions, including no multicollinearity.

### Outcome assessment

Patients with a complicated course had significantly worse outcomes compared with those with an uncomplicated course (Table 2). A complicated course was associated with markedly smaller mean improvement in NIHSS score (multivariate mean difference 5.8, 95% confidence interval [CI]: 4.4–7.1; imputed 5.4, 95% CI: 4.1–6.7), higher odds of pneumonia (multivariate odds ratios [OR]: 3.14, 95% CI: 1.83–5.36; imputed OR: 2.72, 95% CI: 1.66–4.48), poor functional outcome at 3 months (mRS 3–6; multivariate OR: 4.35, 95% CI: 2.47–7.86; imputed OR 3.65, 95% CI 2.15–6.20), and higher 3-month mortality (multivariate OR: 3.94, 95% CI: 2.16–7.22; imputed OR: 4.20, 95% CI: 2.45–7.21).

### Physiology

Descriptive physiological data at PACU admission and discharge for patients with an uncomplicated course are presented in Supplementary Table 4.

## Discussion

In this single-centre retrospective cohort of 734 patients undergoing EVT for AIS under GA with early extubation, anaesthesia-related complications occurred in 133 (18%) cases within 24 h, resulting in a complicated course that most often required direct ICU transfer (*n*=79; 11%). Patients with a complicated course had significantly poorer functional outcomes at 3 months compared with those with an uncomplicated course. In contrast, 82% of patients experienced no complications and had an uncomplicated course, characterised by successful extubation, an uneventful PACU stay, and transfer to the stroke ward without anaesthesia-related complications within 24 h post-EVT. Importantly, the factors defining a complicated *vs* uncomplicated course are heterogeneous, as this category combines neurological deterioration, medical complications, and organisational factors, and therefore captures early postoperative deterioration rather than anaesthesia-specific events.

A complicated course was primarily predicted by markers of clinical severity, including higher admission NIHSS, younger age, and posterior circulation occlusion. These findings are consistent with a previous, smaller study identifying age, NIHSS, and successful reperfusion as significant predictors of unfavourable outcomes after EVT.^17^ Not surprisingly, NIHSS was shown to have predictive value because patients with high NIHSS scores may have a higher risk of postoperative complications independent of anaesthesia. Limitations of our analysis include the limited power and that intraprocedural anaesthesia-related factors such as hypotension, vasopressor requirements, ventilation settings, and BP variability were not assessed because of insufficient data. These factors may have helped to understand the relative contributions of anaesthesia management *vs* intrinsic disease severity to postinterventional complications. Furthermore, a complicated course is not necessarily attributable to GA alone; studies using CS have also reported complications requiring ICU admission. This is reflected by studies evaluating conversion from CS to GA during EVT, where approximately 11%–25% of patients required conversion, often followed by ICU admission and mechanical ventilation.^7^^,^^31^^,^^32^

Extubation failure requiring ICU admission was the most frequent complication, occurring in 9% of all patients.

About half of these cases involved patients intubated prehospital—primarily for posterior circulation stroke—who were not candidates for immediate extubation, while the remainder involved patients intubated during EVT who were not extubated because of neurological impairment or procedural complications. Comparative data on extubation failure after EVT under GA are scarce, and reports of delayed extubation in previous trials likely reflect institutional practice rather than true failure.^9^

Optimal timing of extubation after EVT remains undefined.^4^ Some centres routinely delay extubation for up to 72 h, citing concerns about airway protection, haemodynamic instability, and aspiration risk.^9^^,^^16^, ^17^, ^18^^,^^21^ However, retrospective data suggest that delayed extubation and prolonged ventilation are associated with worse outcomes and increased pneumonia rates.^16^, ^17^, ^18^ In our cohort, pneumonia occurred in 19%, comparable to rates observed in the AMETIS trial and the SAGA meta-analysis, regardless of anaesthesia strategy.^7^^,^^33^

The role of GA in EVT remains debated.^6^, ^7^, ^8^, ^9^^,^^33^, ^34^, ^35^ In our cohort, the early-extubation GA protocol was associated with favourable 3-month functional outcomes (mRS 0–2) in 51% of patients, including those with posterior circulation strokes, which typically carry a poorer prognosis. Although our findings are based on a retrospective analysis and do not compare different anaesthetic strategies, the results are comparable to those from recent multicentre trials, a meta-analysis of single-centre RCTs, and a large retrospective study (Supplementary Table 5), all of which reported similar admission NIHSS scores.^7^^,^^33^, ^34^, ^35^

Functional outcome is strongly associated with reperfusion quality.^33^, ^34^, ^35^ Successful reperfusion (mTICI 2b–3) was achieved in 93% of patients, with short puncture-to-reperfusion times. Although mTICI scoring is subjective, immobilisation achieved under GA may contribute to high reperfusion rates, consistent with prior studies reporting higher recanalisation success with GA than with non-GA approaches.^6^^,^^7^^,^^33^^,^^34^

In this EVT-GA protocol, patients were monitored in the PACU for 2 h before transfer to the stroke ward. Patients with an uncomplicated course showed stable parameters (e.g. oxygen saturation, SBP, and DBP) immediately after extubation. No standardised post-EVT monitoring guidelines exist regarding the level of monitoring or PACU stay duration.^4^

In our cohort, 1.6% of patients required ICU transfer from the PACU because of haemodynamic issues, and 4% needed extended PACU observation before transfer to the stroke ward. Of those discharged to the stroke ward, 1.5% later developed complications requiring ICU transfer. Although no comparative data are available, these findings suggest that a standardised GA protocol for EVT in patients with AIS and high comorbidity results in only minimal load on the PACU and ICU because of post-EVT complications.

Several limitations should be acknowledged. First, this was a retrospective single-centre study without external validation, which limits the generalisability of the findings, particularly in settings with different logistical structures, staffing models, or anaesthesia practices.

At our institution, a specific organisational setup involving highly specialised neuroanaesthesia teams is in place, which may not reflect practice at other centres. Second, all patients with any deviation from the protocol were classified as having a complicated course. This composite endpoint may have introduced heterogeneity and weakened interpretability, as patients with minor deviations (e.g. mild respiratory issues in PACU) were grouped together with those experiencing severe complications requiring ICU transfer. Alternative classification approaches were not feasible because of the limited number of patients within subgroups. Third, the predictive risk may be overstated in patients with high NIHSS scores, as more severe strokes, reflected by higher NIHSS scores or posterior circulation occlusions, may be associated with an increased risk of postinterventional complications independent of anaesthesia. Fourth, the study does not provide external validation, which limits the generalizability to other cohorts of EVT patients. Finally, selection bias may be present, as excluding patients unfit for GA likely removed higher-risk cases. Missing-data bias may also have influenced the results.

In conclusion, anaesthesia-related complications occurred in 18% of cases and were primarily associated with younger age, higher admission NIHSS scores, and posterior circulation occlusions. Patients with a complicated course had significantly poorer functional outcomes at 3 months compared with those with an uncomplicated course. The proportion of patients with favourable functional outcomes in our cohort was consistent with that reported in multicentre studies comparing GA and non-GA approaches and may support the continued use of this protocol in current EVT practice.

## Authors’ contributions

Conception and design of the study: IHJ, AGD, JBV, MR.

Analysis and interpretation of the data: IHJ, AGD, JBV, MR.

Drafting the manuscript and revising it critically for important intellectual content: MR, IHJ, AGD, JBV.

Acquisition of the data: IHJ, MR, ABB, SMS, RAB, RM, CZS.

Revising the manuscript: All authors.

Final approval of the version to be submitted: All authors.

## Declaration of generative artificial intelligence (AI) in scientific writing

During the preparation of this work the author(s) used (Chat GPT 5 / OpenAI) in order to improve readability and language. After using this tool/service, the author(s) reviewed and edited the content as needed and take(s) full responsibility for the content of the publication.

## Funding

MR is supported by the Independent Research Fund Denmark (grant ID: 10.46540/5243-00012B).

CZS is supported by a research grant from Health Research Foundation of Central Denmark Region (grant ID: A3130).

## Declaration of interests

The authors declare that they have no conflict of interest.
