# Supplementary material for: Anaesthesia-related complications after endovascular therapy for acute ischaemic stroke under general anaesthesia with early extubation: A single-centre retrospective cohort study
Source: BJA Open. 2026 Apr 10;18:100547. doi: 10.1016/j.bjao.2026.100547 (PMC13091780; doi:10.1016/j.bjao.2026.100547)

**SUPPLEMENTARY MATERIAL**

**Supplementary Table 1. Factors for absence of post-EVT extubation in pre-procedurally intubated patients.**

| **Factor** | **Number of patients, n** |
| --- | --- |
| **Neurological concern*** | 17 |
| **Haemodynamic instability** | 1 |
| **Respiratory insufficiency** | 6 |
| **Procedure complication**** | 12 |
| **Other causes** | 1 |

**Low level of consciousness prior to intubation.*

***Vessel perforation; groin haemorrhage.*

**Supplementary Table 2. Missing data analysis**

| **Characteristics** | **Complicated**  N = 133 | **Uncomplicated**  N = 601 |
| --- | --- | --- |
| **Age** | 0 (0%) | 0 (0%) |
| **Female sex** | 0 (0%) | 0 (0%) |
| **Admission NIHSS** | 6 (4.5%) | 14 (2.3%) |
| **Pre-stroke mRS** | 0 (0%) | 13 (2.2%) |
| **mrs_pre_num** | 0 (0%) | 13 (2.2%) |
| **Previous AIS** | 2 (1.5%) | 20 (3.3%) |
| **Previous TIA** | 0 (0%) | 10 (1.7%) |
| **Hypertension** | 2 (1.5%) | 9 (1.5%) |
| **Diabetes** | 1 (0.8%) | 11 (1.8%) |
| **Atrial fibrillation** | 1 (0.8%) | 10 (1.7%) |
| **Previous AMI** | 2 (1.5%) | 14 (2.3%) |
| **PAD** | 0 (0%) | 12 (2.0%) |
| **Smoking** | 24 (18%) | 77 (13%) |
| **High alcohol consumption** | 27 (20%) | 70 (12%) |
| **Living alone** | 4 (3.0%) | 9 (1.5%) |
| **Vascular location of embolus** | 1 (0.8%) | 2 (0.3%) |
| **Treated with IV tPA** | 0 (0%) | 0 (0%) |
| **mTICI classification** | 2 (1.5%) | 13 (2.2%) |
| **mTICI 2b-3** | 2 (1.5%) | 13 (2.2%) |
| **mRS at 3 months** | 3 (2.3%) | 14 (2.3%) |
| **mrs_3mdr_num** | 3 (2.3%) | 14 (2.3%) |
| **3 months mRS 3-6** | 3 (2.3%) | 14 (2.3%) |
| **24H NIHSS** | 25 (19%) | 46 (7.7%) |
| **Pneumonia treatment** | 3 (2.3%) | 2 (0.3%) |
| **Time at PACU (hours)** | 79 (59%) | 0 (0%) |
| **Time at ICU (hours)** | 54 (41%) | 601 (100%) |
| **Time from artery puncture to reperfusion (minutes)** | 32 (24%) | 114 (19%) |
| **Time from arrival to reperfusion (minutes)** | 40 (30%) | 139 (23%) |
| **Onset to angiosuite (hours)** | 13 (9.8%) | 65 (11%) |
| **Onset to reperfusion (minutes)** | 30 (23%) | 106 (18%) |
| **Time from MRI arrival to angio suite arrival (minutes)** | 47 (35%) | 180 (30%) |
| **Time from angio suite arrival to artery puncture (minutes)** | 21 (16%) | 79 (13%) |
| **Time from artery puncture to procedure termination (minutes)** | 17 (13%) | 71 (12%) |
| **Angio suite to reperfusion (minutes)** | 29 (22%) | 101 (17%) |
| **Angio suite to end of procedure (minutes)** | 13 (9.8%) | 65 (11%) |
| **Hospital admission (days)** | 15 (11%) | 71 (12%) |

Missing data for baseline characteristic and outcome variables [n (%)].

*Abbreviations: NIHSS = National Institutes of Health Stroke Score; mRS = Modified Rankin Scale; AIS = Acute Ischemic Stroke; TIA = Transient Ischemic Attack; AMI = Acute Myocardial Infarction; PAD = Peripheral Artery Disease; IV tPA = Intravenous Thrombolysis; EVT = Endovascular Therapy; mTICI = modified Thrombolysis in Cerebral Infarction score; PACU = Post-anaesthesia Care Unit; ICU = Intensive Care Unit; MRI = Magnetic Resonance Imaging.*

**Supplementary Table 3. Prediction model regression coefficients.**

|  | **Univariate** | | **Multivariate** | **Multivariate imputed** |
| --- | --- | --- | --- | --- |
| **Characteristic** | **N** | **OR** **(95% CI)** | **OR** **(95% CI)** | **OR** **(95% CI)** |
| **Age** | 734 | 0.98 (0.97 to 1.00) | 0.96 (0.94 to 0.99) | 0.97 (0.95 to 0.98) |
| **Female** | 734 | 1.10 (0.75 to 1.60) | 1.73 (0.99 to 3.04) | 1.41 (0.91 to 2.20) |
| **NIHSS** | 714 | 1.08 (1.05 to 1.11) | 1.08 (1.05 to 1.13) | 1.08 (1.05 to 1.11) |
| **Pre-stroke mRS 3-5** | 721 | 3.24 (1.53 to 6.70) | 3.51 (1.10 to 10.5) | 3.09 (1.31 to 7.26) |
| **Previous AIS** | 712 | 1.09 (0.60 to 1.88) | 0.78 (0.32 to 1.74) | 0.86 (0.44 to 1.66) |
| **Previous TIA** | 724 | 0.65 (0.24 to 1.46) | 0.80 (0.22 to 2.27) | 0.89 (0.35 to 2.27) |
| **Arterial hypertension** | 723 | 1.42 (0.96 to 2.12) | 1.89 (1.02 to 3.61) | 1.49 (0.91 to 2.45) |
| **Diabetes mellitus** | 722 | 1.36 (0.81 to 2.21) | 1.37 (0.63 to 2.86) | 1.17 (0.64 to 2.14) |
| **Atrial fibrillation** | 723 | 0.75 (0.48 to 1.15) | 0.88 (0.43 to 1.74) | 1.06 (0.62 to 1.80) |
| **Previous AMI** | 718 | 0.97 (0.49 to 1.77) | 0.93 (0.38 to 2.12) | 1.08 (0.53 to 2.20) |
| **PAD** | 722 | 1.43 (0.56 to 3.27) | 0.86 (0.24 to 2.72) | 1.18 (0.43 to 3.23) |
| **Smoking** | 633 |  |  |  |
| Never |  | — | — | — |
| Smoking |  | 1.65 (0.98 to 2.78) | 1.74 (0.88 to 3.45) | 1.47 (0.80 to 2.72) |
| Previous |  | 1.66 (1.01 to 2.75) | 1.59 (0.81 to 3.12) | 1.74 (0.94 to 3.22) |
| **High alcohol consumption** | 637 | 1.26 (0.64 to 2.34) | 1.33 (0.56 to 2.93) | 1.25 (0.62 to 2.52) |
| **Living alone** | 721 | 0.74 (0.48 to 1.14) | 0.69 (0.36 to 1.29) | 0.87 (0.52 to 1.44) |
| **Onset to angiosuite (hours)** | 656 | 1.00 (0.99 to 1.00) | 1.00 (0.99 to 1.00) | 1.00 (0.99 to 1.00) |
| **Location of vascular occlusion** | 731 |  |  |  |
| Anterior |  | — | — | — |
| None |  | 1.00 (0.34 to 2.45) | 2.94 (0.84 to 8.95) | 1.16 (0.42 to 3.20) |
| Posterior |  | 4.84 (3.02 to 7.75) | 3.36 (1.63 to 6.89) | 4.78 (2.73 to 8.35) |
| **IV tPA before EVT** | 734 | 0.65 (0.44 to 0.95) | 0.59 (0.33 to 1.03) | 0.77 (0.49 to 1.20) |
| **mTICI 2b-3** | 719 | 0.39 (0.21 to 0.73) | 0.29 (0.12 to 0.71) | 0.34 (0.16 to 0.70) |

*Abbreviations: NIHSS = National Institutes of Health Stroke Score; mRS = modified Rankin Scale; AIS = Acute Ischaemic Stroke; TIA = Transient Ischemic Attack; AMI = Acute Myocardial Infarction; PAD = Peripheral Artery Disease;
IV tPA = Intravenous Thrombolysis; mTICI = modified Thrombolysis in Cerebral Infarction score; 95% CI = 95% Confidence Interval; OR = Odds Ratio.*

**Supplementary Table 4. Physiological measures in PACU of all patients with an uncomplicated course (n = 601).**

|  | **Arrival at PACU** | **Before transfer from PACU** |
| --- | --- | --- |
| **SpO_2_, %** | 98.00 (96.00, 100.00) | 98.00 (97.00, 100.00) |
| **Systolic BP, mmHg, median (IQR), mmHg** | 135 (120, 150) | 130 (120, 145) |
| **Diastolic BP, mmHg, median (IQR), mmHg** | 75 (65, 85) | 70 (65, 80) |
| **MABP, mmHg, median (IQR), mmHg** | 95 (85, 105) | 92 (83, 102) |
| **Oxygen requirement in PACU, median (IQR), L/min** | 2.00 (1.00, 2.50) | |

*Abbreviations: IQR = Interquartile Range; SpO_2_ = Peripheral capillary oxygen saturation; BP = Blood pressure;
MABP = Mean Arterial Blood Pressure; PACU = Post-anaesthesia Care Init; L/min = Litre per minute.*

**Supplementary Table 5. Selected baseline and outcome data from our analysis in the context of recent multicentre randomised trials (AMETIS, GASS) and meta-analyses (SAGA, HERMES) comparing general anaesthesia and conscious sedation in EVT for acute ischemic stroke.**

|  | **Aarhus EVT- GA Protocol** | **AMETIS RCT**  **GA CS** | | **GASS RCT**  **GA CS** | | **SAGA metanalysis**  **GA CS** | | **HERMES metaanalysis**  **GA CS** | |
| --- | --- | --- | --- | --- | --- | --- | --- | --- | --- |
| **Number of patients, n** | 734 | 135 | 138 | 174 | 177 | 183 | 185 | 236 | 561 |
| **Admission NIHSS,**  **Median (IQR)/mean(SD)*** | 15 (8, 19) | 16  (11, 20) | 15  (11, 20) | 16 (± 6)* | 16 (± 5)* | 18 (14, 21) | 17 (14, 20) | 18 (15, 21) | 17 (14, 20) |
| **24-hour NIHSS, Median(IQR/mean(SD)*** | 7 (3, 16) | 9 (3, 19) | 8 (3, 17) | 11 (± 9)* | 11 (± 7)* | 11 (6, 16) | 12 (7, 16) | Na | Na |
| **3-month mRS 0-2, n (%)** | 365 (51) of 717 | 45 (33) | 54 (39) | 66 (40) of 166 | 63 (36) of 175 | 90 (49) | 65 (35) | 94 (40) of 234 | 282 (50) |
| **mTICI 2b-3, n (%)** | 670 (93) of 719 | 115 (85) | 107 (78) | 144 (85) of 169 | 131 (75) of 174 | 133 (73) | 117 (63) | 160 (75) of 213 | 386 (76) of 507 |
| **Pneumonia, n (%)** | 138 (19) of 729 | 26 (19) | 28 (20) | Na | Na | 34 (19) | 36 (20) | 27 (11) | 47 (8) |
| **Time from angiosuite arrival to artery puncture, median (IQR), minutes** | 17 (14, 20) | 11  (8, 18) | 9  (4, 15) | Na | Na | 23 (14, 30) | 15 (10, 24) | Na | Na |
| **Time from artery puncture to reperfusion, median (IQR), minutes** | 24 (14, 41) | 35  (25, 58) | 41  (24, 62) | Na | Na | 51.5 (31, 90) | 70.5 (34, 105) | Na | Na |
| **Time from stroke onset to reperfusion, Median(IQR), minutes** | 276  (185, 524) | Na | Na | 320 (±96)* | 307 (± 87)* | 241.5 (200, 349) | 257 (197, 325) | 302 (246, 357) | 288 (222, 358) |

*Abbreviations: IQR = Interquartile Range; SD = Standard Deviation; NIHSS = National Institutes of Health Stroke Score; mRS = modified Rankin Scale; mTICI = modified Thrombolysis in Cerebral Infarction score; EVT = Endovascular Therapy; GA = General Anaesthesia; CS = Conscious Sedation; RCT = Randomised Clinical Trial.*

**Supplementary Figure 1. ROC curves illustrating model performance in predicting a complicated course.**


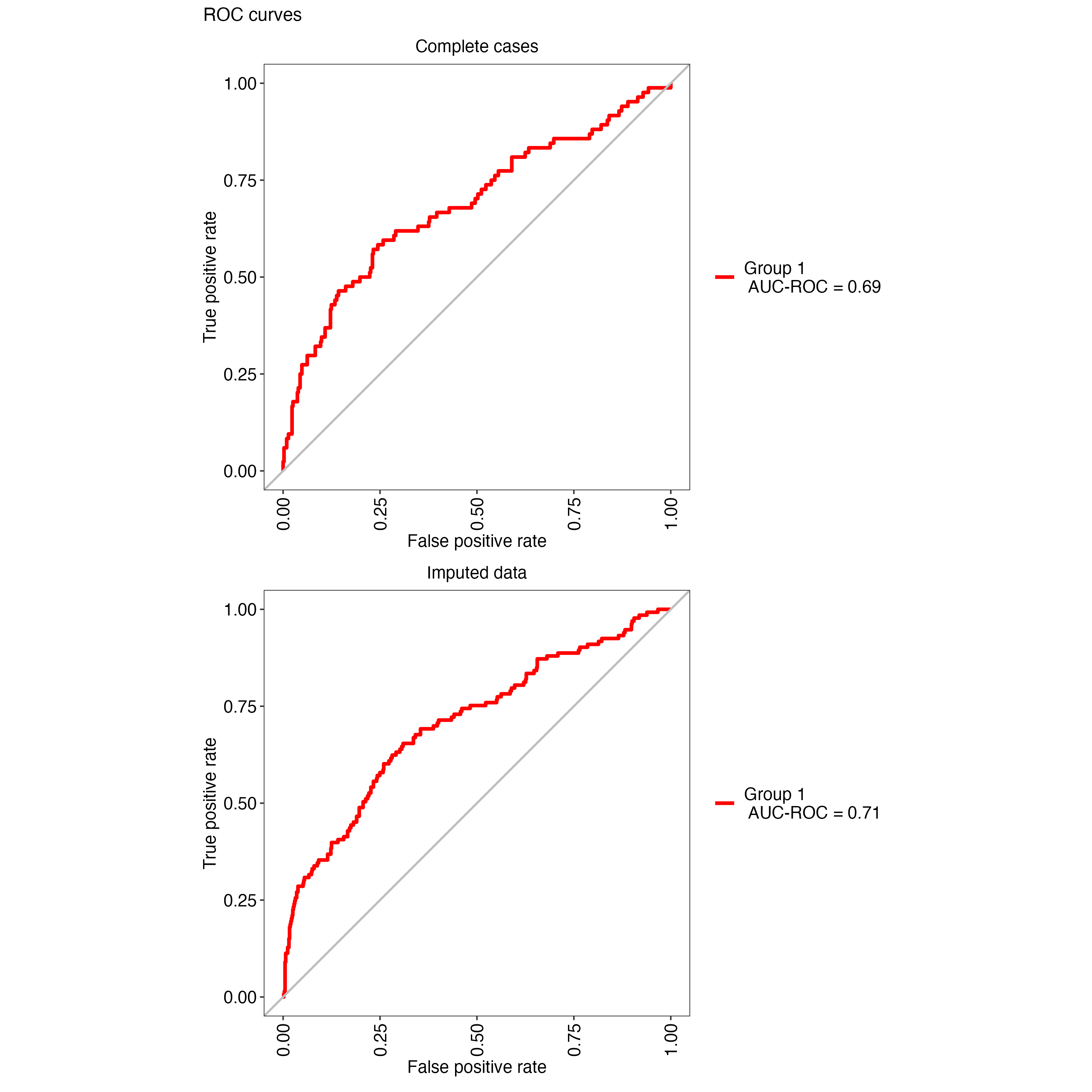


Panel A (upper) shows the ROC curve for the observed dataset (AUC = 0.69), and Panel B (lower) for the imputed dataset (AUC = 0.71). Both values indicate moderate discriminative ability.

*Abbreviations: ROC = Receiver Operating Characteristic; AUC = Area Under the Curve.*

**Supplementary Figure 2. Calibration plots for observed (upper) and imputed (lower) data sets.**


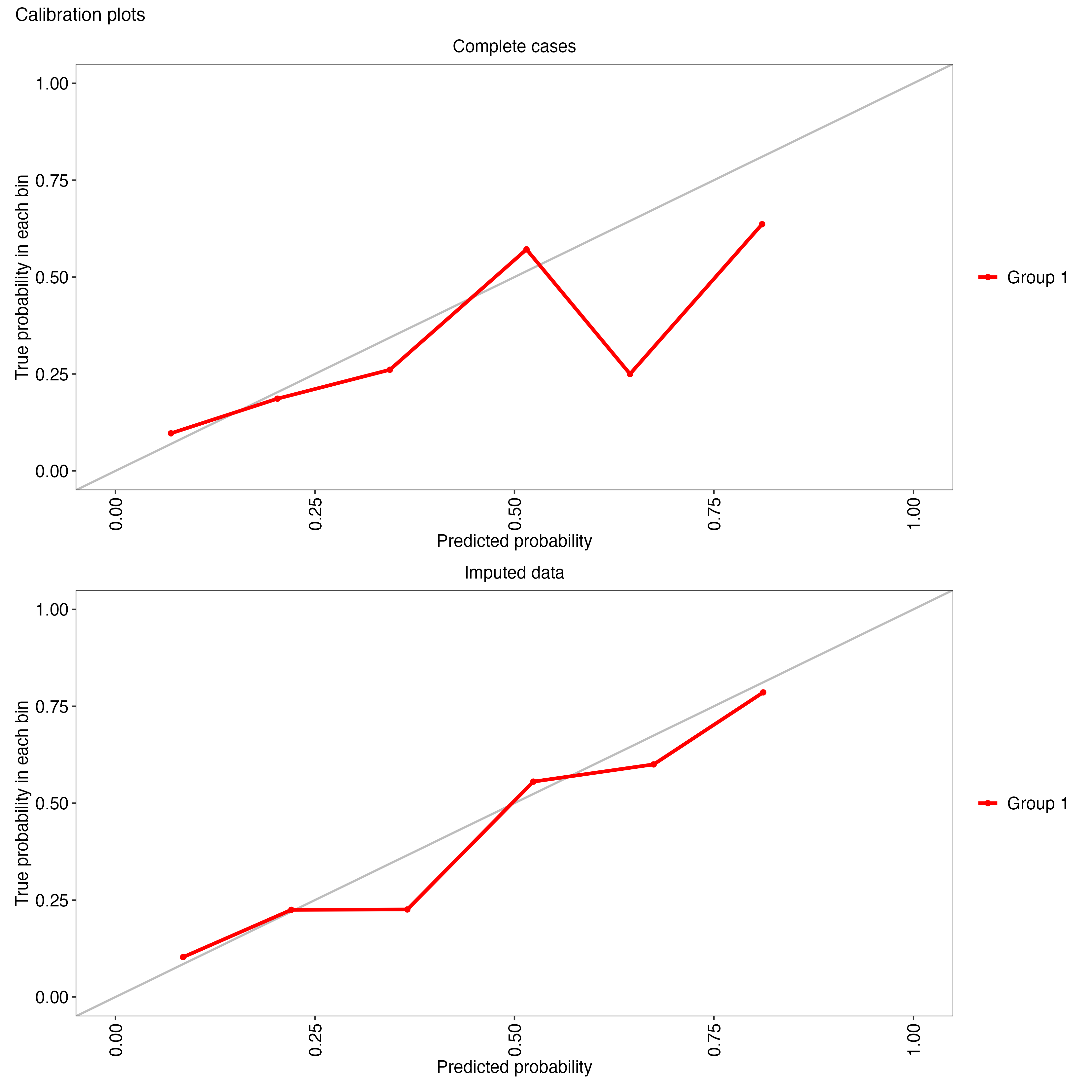

Supplement: Multimedia component 1 [file mmc1.docx]
